# Supplementary material for: Expression of Prostaglandin Genes and β-Catenin in Whole Blood as Potential Markers of Muscle Degeneration
Source: Int J Mol Sci. 2023 Aug 17;24(16):12885. doi: 10.3390/ijms241612885 (PMC10454559; doi:10.3390/ijms241612885)
Supplement: Supplementary file 1 [file ijms-24-12885-s001.zip › ijms-2565838-supplementary.pdf]

International Journal of
Molecular Sciences

MDPI

Supplement

Table S1. Missing data in analyzed sarcopenia, frailty syndrome and geriatric patients (geriatric control) groups.

Analyzed parameter	Missing data N=73 n (%)	Sarcopenia N =12 n (%)	Frailty syndrome N = 36 n (%)	Geriatric control N = 25 n (%)
Expression of *PTGES2*	0 (0%)	0 (0%)	0 (0%)	0 (0%)
Expression of *PTGS2*	0 (0%)	0 (0%)	0 (0%)	0 (0%)
Expression of *PTGER4*	0 (0%)	0 (0%)	0 (0%)	0 (0%)
Expression of *CTNNB1*	0 (0%)	0 (0%)	0 (0%)	0 (0%)
Age (age)	0 (0%)	0 (0%)	0 (0%)	0 (0%)
gender	0 (0%)	0 (0%)	0 (0%)	0 (0%)
FI-CGA	0 (0%)	0 (0%)	0 (0%)	0 (0%)
TUG (s)	7 (10%)	1 (8%)	3 (8%)	3 (12%)
ASMM (kg)	33 (45%)	0 (0%)	22 (61%)	11 (44%)
IL-1β (pg/ml)	3 (4%)	0 (0%)	1 (3%)	2 (8%)
IL-6 (pg/ml)	3 (4%)	0 (0%)	1 (3%)	2 (8%)
TNF-α (pg/ml)	3 (4%)	0 (0%)	1 (3%)	2 (8%)
IFN-γ (pg/ml)	3 (4%)	0 (0%)	1 (3%)	2 (8%)
IL-1α (pg/ml)	3 (4%)	0 (0%)	1 (3%)	2 (8%)
IL-10 (pg/ml)	4 (5%)	0 (0%)	1 (3%)	2 (8%)
LDH (U/L)	8 (11%)	6 (50%)	1 (3%)	1 (4%)
CK (U/L)	2 (0.03)	1 (8%)	0 (0%)	1 (4%)
Osteopenia	0 (0%)	0 (0%)	0 (0%)	0 (0%)
Osteoporosis	0 (0%)	0 (0%)	0 (0%)	0 (0%)
Polyarthritis	0 (0%)	0 (0%)	0 (0%)	0 (0%)
Degenerative Disc Disease	0 (0%)	0 (0%)	0 (0%)	0 (0%)
Vit. D3 (ng/ml)	1 (1%)	0 (0%)	1 (3%)	0 (0%)
NT-proBNP (pg/ml)	11 (15%)	0 (0%)	5 (14%)	6 (24%)
Serum Fe level (mcg/dL)	43 (59%)	6 (50%)	19 (53%)	18 (72%)
Vit. B12 (pg/mL)	0 (0%)	0 (0%)	0 (0%)	0 (0%)
ESR (mm/h)	0 (0%)	0 (0%)	0 (0%)	0 (0%)
CRP (mg/dL)	0 (0%)	0 (0%)	0 (0%)	0 (0%)
Albumines (g/dl)	1 (1%)	0 (0%)	1 (3%)	0 (0%)
Decrease of albumines	1 (1%)	0 (0%)	1 (3%)	0 (0%)
Hemoglobine (g/dl)	0 (0%)	0 (0%)	0 (0%)	0 (0%)
Creatinine (μmol/L)	0 (0%)	0 (0%)	0 (0%)	0 (0%)
Cholesterol (mg/dL)	1 (1%)	0 (0%)	1 (3%)	0 (0%)
LDL (mg/dL)	1 (1%)	0 (0%)	1 (3%)	0 (0%)
Triglycerides (mm/l)	1 (1%)	0 (0%)	1 (3%)	0 (0%)
BMI (kg/m^2)	1 (1%)	0 (0%)	1 (3%)	0 (0%)

FI-CGA - Frailty Index Based on a Comprehensive Geriatric Assessment; TUG- Timed up-and-go scale (mobility scale); ASMM- appendicular skeletal muscle mass; LDH- Lactate Dehydrogenase ; CK- creatine phosophokinase; NT-proBNP - N-terminal (NT)-pro hormone BNP; Fe- iron; ESR- erythrocyte sedimentation rate; CRP- C-reactive protein; LDL - low-density lipoprotein; BMI – body mass index

Table S2. Post hoc analysis of significant differences in analyzed groups.

parameter	Pairwise post hoc P value Bonferroni adjusted		
	**Geriatric control - Frailty syndrome**	**Geriatric control – Sarcopenia**	**Frailty syndrome - Sarcopenia**
ASMM (kg)	0.39	<0.001	<0.001
Serum Fe level (mcg/dL)	<0.001	0.82	0.04
Age (years)	0.006	0.46	0.95
FI-CGA	<0.001	0.04	<0.001
BMI	0.27	0.003	0.05
LDL	0.004	0.23	0.68
Cholesterol (mg/dL)	0.02	0.79	0.31
Hemoglobine (g/dl)	0.02	0.35	0.74
TUG (s)	0.002	0.38	0.18
ESR (mm/h)	0.14	0.48	0.09

ASMM- appendicular skeletal muscle mass; Fe- iron; FI-CGA - Frailty Index Based on a Comprehensive Geriatric Assessment; BMI – body mass index; LDL - low-density lipoprotein; TUG- Timed up-and-go scale (mobility scale); ESR- erythrocyte sedimentation rate

Table S3. Frequency of the patients in the analyzed groups in categorized Body Mass Index (BMI).

	**frailty syndrome (N = 36)**	**geriatric control (N = 25)**	**sarcopenia (N = 12)**
underweight	2 (5.56%)	0 (0.00%)	1 (8.33%)
normal weight	13 (36.11%)	4 (16.00%)	6 (50.00%)
overweight	7 (19.44%)	9 (36.00%)	5 (41.67%)
obesity	14 (38.89%)	12 (48.00%)	0 (0.00%)

Table S4. Summary of ELISA test and below the limit of detection.

	**25-30 (N = 51)**	**>50 (N = 42)**	**geriatric control (N = 25)**	**frailty syndrome (N = 36)**	**sarcopenia (N = 12)**
Missing data n	10	1	2	1	0
**IL-1β level**					
below the limit of detection < 10.42	30 (73.17%)	37 (90.24%)	15 (65.22%)	14 (40.00%)	6 (50.00%)
measured	11 (26.83%)	4 (9.76%)	8 (34.78%)	21 (60.00%)	6 (50.00%)
**IFN-y level**					
below the limit of detection < 20.86	38 (92.68%)	41 (100.00%)	22 (95.65%)	32 (91.43%)	12(100.00%)
measured	3 (0.00%)	0 (0.00%)	1 (4.35%)	3 (8.57%)	0 (0.00%)
**IL-1 level**					
below the limit of detection < 20.86	38 (92.68%)	41 (100.00%)	23 (100.00%)	33 (94.29%)	12(100.00%)
measured	3 (7.32%)	0 (0.00%)	0 (0.00%)	2 (5.71%)	0 (0.00%)
**IL-10 level**					

below the limit of detection < 20.86	36 (87.80%)	40 (97.56%)	16 (69.57%)	19 (55.88%)	8 (66.67%)
measured	5 (12.20%)	1 (2.44%)	7 (30.43%)	15 (44.12%)	4 (33.33%)
**TNF-α level**					
below the limit of detection < 7.96	20 (48.78%)	11 (26.83%)	0 (0.00%)	0 (0.00%)	0 (0.00%)
measured	21 (51.22%)	30 (73.17%)	23 (100.00%)	35 (100.00%)	12(100.00%)
